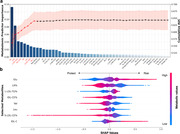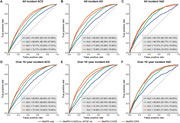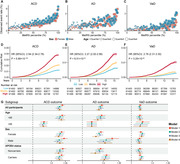# Plasma metabolic profiles predict future dementia and dementia subtypes: a prospective analysis of 274,160 participants

**DOI:** 10.1002/alz.088149

**Published:** 2025-01-09

**Authors:** Xiao‐Yu He, Yi‐Xuan Qiang, JIA YOU, Wei Cheng, Jin‐Tai Yu

**Affiliations:** ^1^ Huashan Hospital, Fudan University, Shanghai China; ^2^ Shanghai Medical College, Fudan University, Shanghai, Shanghai China; ^3^ Fudan University, Shanghai China; ^4^ Institute of Science and Technology for Brain‐Inspired Intelligence, Fudan University, Shanghai, Shanghai China; ^5^ National Center for Neurological Disorders, Shanghai China

## Abstract

**Background:**

Blood‐based biomarkers for dementia are gaining attention due to their non‐invasive nature and feasibility in regular healthcare settings. Here, we explored the associations between 249 metabolites with all‐cause dementia (ACD), Alzheimer's disease (AD), and vascular dementia (VaD) and assessed their predictive potential.

**Method:**

This study included 274,160 participants from the UK Biobank. Cox proportional hazard models were employed to investigate longitudinal associations between metabolites and dementia. The importance of these metabolites was quantified using machine learning algorithms, and a metabolic risk score (MetRS) was subsequently developed for each dementia type. We further investigated how MetRS stratified the risk of dementia onset and assessed its predictive performance, both alone and in combination with demographic and cognitive predictors.

**Result:**

During a median follow‐up of 14.01 years, 5,274 participants developed dementia. Of the 249 metabolites examined, 143 were significantly associated with incident ACD, 130 with AD, and 140 with VaD. Among metabolites significantly associated with dementia, lipoprotein lipid concentrations, linoleic acid, sphingomyelin, glucose, and branched‐chain amino acids ranked top in importance. Individuals within the top tertile of MetRS faced a significantly greater risk of developing dementia than those in the lowest tertile. When MetRS was combined with demographic and cognitive predictors, the model yielded the area under the receiver operating characteristic curve (AUC) values of 0.857 for ACD, 0.861 for AD, and 0.873 for VaD.

**Conclusion:**

We conducted the largest metabolome investigation of dementia to date, for the first time revealed the metabolite importance ranking, and highlighted the contribution of plasma metabolites for dementia prediction.